# Loss of seryl-tRNA synthetase (*SARS1*) causes complex spastic paraplegia and cellular senescence

**DOI:** 10.1136/jmg-2022-108529

**Published:** 2022-08-30

**Authors:** Edgard Verdura, Bruno Senger, Miquel Raspall-Chaure, Agatha Schlüter, Nathalie Launay, Montserrat Ruiz, Carlos Casasnovas, Agustí Rodriguez-Palmero, Alfons Macaya, Hubert Dominique Becker, Aurora Pujol

**Affiliations:** 1 Neurometabolic Diseases Laboratory, Bellvitge Biomedical Research Institute (IDIBELL), L'Hospitalet de Llobregat, 08908, Barcelona, Catalonia, Spain; 2 Centre for Biomedical Research on Rare Diseases (CIBERER), Instituto de Salud Carlos III, Madrid, Spain; 3 Université de Strasbourg 1, Strasbourg, France; 4 Pediatric Neurology Research Group, Vall d’Hebron University Hospital, Universitat Autònoma de Barcelona, 08035, Barcelona, Catalonia, Spain; 5 Department of Paediatric Neurology, Vall d’Hebron University Hospital, 08035, Barcelona, Catalonia, Spain; 6 Hospital Universitari de Bellvitge, Barcelona, Spain; 7 Pediatrics, Hospital Germans Trias i Pujol, Barcelona, Spain; 8 Institut de Neurociències, Universitat Autònoma de Barcelona, 08193, Barcelona, Catalonia, Spain; 9 Catalan Institution of Research and Advanced Studies (ICREA), 08010, Barcelona, Catalonia, Spain

**Keywords:** sequence analysis, RNA, pediatrics, neurology, nervous system diseases, genetic research

## Abstract

**Background:**

Aminoacyl-tRNA synthetases (ARS) are key enzymes catalysing the first reactions in protein synthesis, with increasingly recognised pleiotropic roles in tumourgenesis, angiogenesis, immune response and lifespan. Germline mutations in several ARS genes have been associated with both recessive and dominant neurological diseases. Recently, patients affected with microcephaly, intellectual disability and ataxia harbouring biallelic variants in the seryl-tRNA synthetase encoded by seryl-tRNA synthetase 1 (*SARS1*) were reported.

**Methods:**

We used exome sequencing to identify the causal variant in a patient affected by complex spastic paraplegia with ataxia, intellectual disability, developmental delay and seizures, but without microcephaly. Complementation and serylation assays using patient’s fibroblasts and an *Saccharomyces cerevisiae* model were performed to examine this variant’s pathogenicity.

**Results:**

A *de novo* splice site deletion in *SARS1* was identified in our patient, resulting in a 5-amino acid in-frame insertion near its active site. Complementation assays in *S. cerevisiae* and serylation assays in both yeast strains and patient fibroblasts proved a loss-of-function, dominant negative effect. Fibroblasts showed an abnormal cell shape, arrested division and increased beta-galactosidase staining along with a senescence-associated secretory phenotype (raised interleukin-6, p21, p16 and p53 levels).

**Conclusion:**

We refine the phenotypic spectrum and modes of inheritance of a newly described, ultrarare neurodevelopmental disorder, while unveiling the role of SARS1 as a regulator of cell growth, division and senescence.

**WHAT IS ALREADY KNOWN ON THIS TOPIC**

- Patients affected with microcephaly, intellectual disability and ataxia harbouring biallelic variants in the seryl-tRNA synthetase 1 (*SARS1*) were reported.
- Up to now, recessive variants in aminoacyl-tRNA synthetases (ARS) genes have been predominantly associated with early onset diseases impacting the central nervous system, compared with dominant variants.

**WHAT THIS STUDY ADDS**

- We describe a novel clinical phenotype and mode of inheritance (*de novo* dominant) for a variant in *SARS1* gene, and unveil the role of SARS1 as a regulator of cell growth, division and senescence.

**HOW THIS STUDY MIGHT AFFECT RESEARCH, PRACTICE OR POLICY**

- We underscore the need for caution interpreting variants in ARS enzyme genes according to known inheritance modes, with important implications for genetic diagnosis and counselling.
- Furthermore, we reveal pivotal roles of this enzyme in the control of cell growth and senescence, underlying the pathogenetic mechanisms of these diseases, and open the way to further research.

## Introduction

Aminoacyl-tRNA synthetases (ARS) are ubiquitously expressed and highly evolutionarily conserved enzymes responsible for esterification of amino acids (AA) to their cognate transfer RNA (tRNA), an essential reaction in decoding mRNA into protein.^1^ ARS catalyse a two-step reaction in which the given amino acid is condensed with ATP to form tightly bound aminoacyl adenylate (AA-AMP), with the simultaneous release of pyrophosphate PPi. The activated amino acid is then transferred from AMP to the 3’-end of its cognate tRNA, producing AA-tRNA and AMP.^2^

Mutations in ARS enzymes have been implicated in both recessive and dominant diseases affecting a broad range of tissues.^3^ While recessive inheritance is very frequently associated with severe, early onset diseases which impact the central nervous system and other organs, dominant mutations cause specifically late-onset peripheral neuropathies in a restricted list of cytoplasmic ARS genes.^4^ It is still unclear why mutations in these ubiquitous enzymes cause such a highly specific phenotype. Seryl-tRNA synthetase 1 (SARS1) is a cytoplasmic ARS enzyme that charges serine onto its cognate tRNA^Ser^ but also onto the non-cognate selenocysteine tRNA^Sec^. Subsequently, serine-bound tRNA^Sec^ undergoes tRNA conversion of the bound serine to selenocysteine by a four-step reaction.^5^

SARS1 is essential for vascular development as shown in a zebrafish model,^6^ and also in a rapid network formation assay of human umbilical vein endothelial cells.^7^ This non-canonical function is linked to the UNE-S domain, a unique domain which has only been found in the C-terminus of vertebrate SARS1. UNE-S harbours a nuclear localisation signal and can divert a fraction of the SARS1 protein into the nucleus to control the expression of vascular endothelial growth factor A.^8^

Two recent articles reported homozygous loss-of-function missense mutations in *SARS1* in two independent families exhibiting a neurodevelopmental syndrome including microcephaly, intellectual disability, seizures and ataxia,^9^ together with other anomalies such as cardiomyopathy, deafness and decompensation during fever.^10^ In this work, we identified a *de novo* inherited novel pathogenic variant of *SARS1* causing a complex spastic paraplegia and ataxia phenotype, thus confirming the recent implication of mutations in this gene in neurological and movement disorders.^9 10^ Furthermore, this variant illustrates the concept that dominant negative mutations in ARS genes can give rise to phenotypes similar to those caused by recessive mutations, rather than the neuropathies frequently linked to dominantly inherited variants.

## Materials and methods

### Patients and samples

The patient was recruited and examined at Vall d’Hebron Hospital, Barcelona. Detailed neurological examination was performed. Blood samples and skin-derived fibroblast cell lines were obtained using standard methods. DNA was extracted using the Gentra Puregene Blood Kit (QIAGEN).

### Whole-exome sequencing analysis

Genomic DNA was extracted from peripheral blood using standard methods. Whole-exome sequencing (WES) was performed on patient’s DNA sample using the SureSelect XT Human All Exon V5 50 Mb kit (Agilent) for DNA capture. WES was performed using the HiSeq 2000 Platform (Illumina) at *Centre Nacional d’Anàlisi Genòmica* (Barcelona). Variants were filtered using an in-house pipeline based on GATK best practice guidelines and prioritised based on accurate patient characterisation with Human Phenotype Ontology (HPO) terms, interaction networks at physical and functional levels and variant intolerance scores generated by the Exome Aggregation Consortium (ExAC) and gnomAD consortia. We prioritised non-synonymous coding variants that had a frequency lower than 0.01 in the ExAC, 1000 Genomes and gnomAD control databases. Candidate variants were validated and tested for cosegregation in all family members by Sanger sequencing.

### Evolutionary conservation of SARS1

SARS1 protein orthologues from multiple species were derived from the following GenBank accessions: XP_009425317.1 (*Pan troglodytes*), NP_001191908.1 (*Mus musculus*), NP_001026563.1 (*Gallus gallus*), NP_998852.1 (*Xenopus tropicalis*), NP_001003882.1 (*Danio rerio*), NP_501804.1 (*Caenorhabditis elegans*), NP_309005.1 (*Escherichia coli*), NP_608743.2 (*Drosophila melanogaster*) and NP_010306.1 (*Saccharomyces cerevisiae*). Calculation of amino acid sequence identity with *Homo sapiens* and alignment were performed using Clustal Omega (https://www.ebi.ac.uk/Tools/msa/clustalo/).

### Three-dimensional modelling

The domain structure of the *SARS1* gene was retrieved from the UniProt database and NCBI. The three-dimensional (3D) structure of wild-type (WT) *SARS1* was retrieved from https://www.rcsb.org/, and a model of the mutated *SARS1* was obtained using the SWISS-MODEL utility on the ExPASy webpage (https://swissmodel.expasy.org). Structures were visualised with PyMOL.

### Fibroblast cultures

Patient dermal fibroblasts were generated from a skin biopsy using standard cell culture procedures. Fibroblasts from the patient and age-matched and sex-matched controls were grown in DMEM (Dulbecco's Modified Eagle's Medium)(Ref: 31885-023) containing 1% penicillin/streptomycin and 10% fetal bovine serum (FBS). Cultures were maintained at 37°C under 5% CO_2_ and 95% humidity.

### RT-PCR and qRT-PCR

RNA was extracted from patient and control fibroblasts using the RNeasy Mini Kit (QIAGEN). Total RNA quantity and quality were assessed by 0.8% agarose gel electrophoresis and NanoDrop spectrophotometry. cDNA was synthesised using the SuperScript IV kit (Life Technologies) following the manufacturer’s instructions. cDNA was amplified by PCR using primers designed with Primer3 using the whole coding sequence of SARS1 (NM_001330669) (forward: GGAACAGGCTCTCATCCAGTA; reverse: AGGAGACCAACTCACGGAAG). Products were separated by agarose gel electrophoresis (3%), purified (QIAquick Gel Extraction Kit, QIAGEN) and sequenced by the Sanger method using standard methodology. cDNA PCR products were cloned into pCMV-Tag2 plasmid using standard methods, and several colonies were extracted for DNA and sequenced to isolate WT and mutated sequences. qRT-PCR was performed in a LightCycler 480 device using SYBR Green I Master Mix (Roche). All reactions were performed in triplicate. Each gene mRNA level was normalised to that of *H36B4* using the 2^–∆∆Ct^ method. A two-sided Student’s t-test was used to compare the expression ratios of patients and controls. Data are presented as the mean±SD. All primers are shown in online supplemental table 1.

10.1136/jmg-2022-108529.supp1 Supplementary data

### Immunofluorescence

Fibroblasts from the index patient and three age-matched and sex-matched controls were seeded on coverslips and cultured in a 6-well plate (200 000 per well) at 37°C for 24 hours. Coverslips were fixed with 4% paraformaldehyde, blocked and incubated overnight with mouse anti-α-tubulin (dilution 1/10 000, Ref: ab80779, Abcam) and rabbit anti-SARS1 (dilution 1/10, Ref: ab183025, Abcam) primary antibodies. Slides were incubated with goat antirabbit IgG Alexa Fluor 488 (dilution 1/1000, Ref: A-21428, Dako) and antimouse IgG Alexa Fluor 647 (dilution 1/1000, Ref: A-21236, Dako) secondary antibodies. DAPI (4′,6-diamidino-2-phenylindole) was used to stain cell nuclei. Confocal microscopy images were acquired with a Leica TCS SL laser scanning confocal spectral microscope using a 63× objective. Images were treated using FIJI software.

### Western blot analysis

For western blot analyses, fibroblasts were homogenised in RIPA (Radioimmunoprecipitation assay) buffer (150 mM NaCl, 1% (w/v) Nonidet P40, 0.5% (v/v) sodium deoxycholate, 0.1% sodium dodecyl-sulfate (SDS) (w/v), 50 mM Tris-HCl, pH 8.0), sonicated for 2 min at 4°C, centrifuged, mixed with 4× NuPAGE LDS Sample Buffer (Invitrogen) and heated at 70°C for 10 min. Protein samples (25 μg) were subjected to polyacrylamide gel electrophoresis at 120 V in NuPAGE MOPS SDS Running Buffer (Invitrogen) supplemented with 5 mM sodium bisulfite (Ref: 243973, Sigma-Aldrich). Proteins were transferred to nitrocellulose membranes using an iBlot 2 Gel Transfer Device (Invitrogen). After blocking in 5% bovine serum albumin (BSA, Sigma-Aldrich) and 0.05% TBS-Tween for 1 hour at room temperature, membranes were incubated with primary antibodies overnight at 4°C. The primary antibodies used included mouse anti-β-actin (dilution 1/10 000, Ref: A2228, Sigma-Aldrich); rabbit H2AX (Chomatin Immunoprecipitation grade, dilution 1/1000, Ref: ab11175, Abcam); mouse γ-H2AX S139 (dilution: 1/1000, Ref: ab22551, Abcam), rabbit anti-SARS1 (dilution 1/1000, Ref: ab183025, Abcam) and γ-tubulin (dilution 1/10 000, Ref: T6557, Sigma). After incubation with secondary antibodies for 1 hour at room temperature, proteins were detected with a ChemiDoc Touch Imaging System (Bio-Rad).

### β-Galactosidase staining

Fibroblasts from the index patient and two controls were seeded on coverslips and cultured in a 6-well plate (200 000 per well) at 37°C for 24 hours. Staining was performed following the manufacturer’s instructions (Senescence Cells Histochemical Staining Kit, Sigma). Photos were taken using a Nikon Eclipse 80i microscope, 10× objective and NIS-Elements BR software. We counted a minimum of 50 cells per individual using the FIJI Cell Counter Tool. Experiments were performed twice. A two-sided Student’s t-test was used to compare the proportion of β-galactosidase (β-gal)-positive cells (%) between the patients and controls. Data are presented as the mean±SD.

### Construction, culture and analysis of *S. cerevisiae* yeast strains

The *SES1* open reading frame (ORF) was replaced by either human WT SARS1 (plasmid pRS316-hSARS) or the mutant version of hSARS (plasmid pRS316-hSARSm) using isothermal assembly.^11^ Each plasmid was transformed into the Y23962 yeast strain (EUROSCARF collection), which is heterozygous diploid for SES1 and has the following genotypes: *MATa/MATα; ura3Δ0/ura3Δ0; leu2Δ0/leu2Δ0; his3Δ1/his3Δ1; met15Δ0/MET15; LYS2/lys2Δ0; YDR023w/YDR023w::kanMX4*.

Transformed Y23962 strains were grown on SDC media lacking uracil (SDC-Ura) (6.75 g/L yeast nitrogen base without amino acids, 20 g/L glucose, 0.55 g complete supplemental media and 20 g/L agar) for 12 hours and transferred to sporulation media (10 g/L yeast extract, 20 g/L peptone, 10 g/L potassium acetate pH 6.5, adjusted with 6 N HCl and 20 g/L agar) for 3–5 days. On sporulation, tetrads were dissected on complete YPD(Yeast extract Peptone Dextrose) solid media (10 g/L yeast extract, 20 g/L peptone, 20 g/L glucose and 20 g/L agar), and viable spores were analysed using a Singer dissection microscope on YPD plates. Individual spores were then restreaked on (i) SDC-Ura to test for the presence of the pRS316 plasmid and (ii) YPD+G418 (500 µg/mL) to test for the presence of the disrupted genomic copy of *SES1*. Drop tests were performed by growing yeast strains in SDC-Ura-Leu liquid media to an optical density (OD)_600 nm_ of 1. Serial dilutions were performed in water, and 6 µL of each dilution was spotted on an SDC-Ura-Leu plate and incubated at 30°C. Pictures were taken at various times of incubation.

### In vitro analysis of SARS1 activity

To analyse hSARS serylation activity, we prepared S100 extracts from *S. cerevisiae* yeast and fibroblasts (see *S. cerevisiae* yeast S100 preparation). These crude enzymatic preparations were then used in tRNA aminoacylation assays with ^14^C-radiolabelled serine (Serine, L-[3-14C] PerkinElmer Ref NEC827050UC, Lot 2361822 52.4 mCi/mmol; 50 µCi/mL). All reactions were carried out at 30°C for yeast extracts and 37°C for fibroblast extracts under the following conditions: 50 mM HEPES (4-(2-hydroxyethyl)-1-piperazineethanesulfonic acid), KOH buffer pH 7.5, 20 mM KCl, 15 mM MgCl_2_, 4 mM DTT (Dithiothreitol), 5 mM ATP, 0.1 mg/mL BSA, 440 µM total yeast tRNA and 74 µM of [14C] serine. tRNA concentration was 22 µM. Data were corrected according to the extract’s concentration. Ten microlitre aliquots from a 62 µL reaction mixture were spotted onto Whatman paper and quenched with 5% trichloroacetic acid (TCA) at various time intervals. After three TCA washes, papers were washed three times with ethanol and dried. Radioactivity was measured in a Beckman LS 6500 scintillation counter with a toluene-based scintillant. The reaction times were 2, 4, 8, 16, 24, 32 min for yeast extracts and 2, 8, 60, 180, 360 min for fibroblasts extracts. Experiments were performed five times independently

### *S. cerevisiae* yeast S100 preparation

Cells were grown in 500 mL SDC-leu-Ura on a rotary shaker at 30°C and harvested when OD_600 nm_=1 by centrifugation at 5000× g for 5 min at 4°C. The pellet was resuspended in 5 mL of lysis buffer containing 50 mM Na-HEPES pH 7, 30 mM KCl, 10% glycerol, 0.1 mM EDTA, 5 mM β-mercaptoethanol and protease inhibitors tablet (Roche). One volume of glass beads (Ø 0.25–0.5 mm, Roth) was added and cell lysis was performed with a FastPrep-24 apparatus (6×1 min at 6.5 m/s, with 1 min on ice between each cycle). Cells debris were removed by centrifugation at 500× g for 10 min at 4°C and the supernatant is centrifuged at 100 000× g for 1 hour. The resulting soluble fraction (S100) is recovered, then dialysed O/N against the storage buffer (same as for fibroblasts extracts). Strains used were hSARS, hSARS+hSARSmut or WT (see online supplemental table 2).

### Fibroblast S100 preparation

Fibroblasts were grown in DMEM media supplemented with FBS and antibiotics (streptomycin and penicillin) and harvested at 80% confluence and issued from three 75 cm^2^ flask. They were lysed in the following buffer : 30 mM Na-HEPES pH 7.9, 10 mM KCl, 1.5 mM MgCl_2_, 5 mM β-mercaptoethanol and protease inhibitors tablet (Roche). Lysis was completed in an appropriate douncer with 12 strokes. Cell debris were removed by centrifugation at 4°C at 8 000× g. The supernatant was then centrifuged at 100 000× g for 1 hour. The extracts wad then dialysed against 50 mM Na-HEPES pH 7.5, 30 mM KCl, 50% glycerol, 0.1 mM EDTA and kept at −20°C. Experiments were performed five times independently.

### Data availability

The authors confirm that the data supporting the findings of this study are available within the article and its online supplemental materials.

## Results

### Clinical description and genetic findings

The patient is an early adolescent Caucasian male with no relevant family history, born at term after an uneventful pregnancy and with a normal neonatal period. He started having focal seizures as a toddler, frequently precipitated by fever, which lasted up to 10 min and on some occasions were followed by brief postictal paresis. Seizures did not respond to phenobarbitone or valproate monotherapy but were eventually controlled with combination of valproate and lamotrigine at middle childhood. He remained seizure-free despite AED withdrawal at late childhood. Electroencephalograms were normal or showed infrequent multifocal spikes. Serial brain MRI at the beginning of adolescence showed subtle and non-progressive punctiform frontal subcortical hyperintensities. MRS was normal. Funduscopy and electroretinogram were normal, but visual evoked potentials showed bilateral increased latencies with normal amplitudes.

He had a global developmental delay, with late acquisition of independent walking at early childhood, motor clumsiness and delayed speech, being able to produce only a few bisyllables during early childhood. Early clumsiness evolved to overt signs of spastic paraparesis that worsened slowly during middle childhood but subsequently remained stable. He underwent surgery for strabismus just before early adolescence. The most recent exam showed prominent and symmetrical signs of spasticity in the lower limbs, with hyper-reflexia, bilateral ankle clonus and right Babinski sign, and a wide-base unsteady spastic gait. He showed no dysmorphic traits, and head growth was normal. Relevant complementary exams included repeatedly normal plasma and cerebrospinal fluid amino acid profiles (serine: 125 μM, normal value 89–165; glycine: 236 μM, normal value 147–299; cysteine: 28 μM, normal value 24–54).

**Figure 1:**
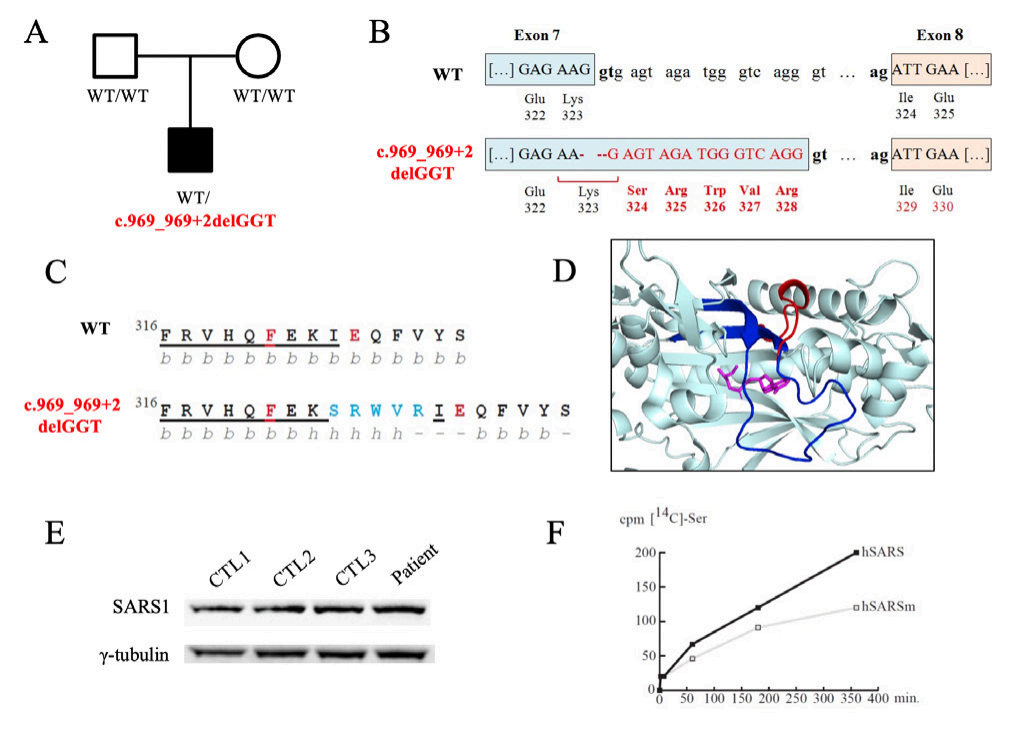
*Seryl-tRNA synthetase 1 (SARS1) de novo* variant features. (A) Family tree. Square: male, circle: female, black symbol: affected individual, white symbols: unaffected individuals, WT: wild-type allele. (B) Representation of variant c.969_969+2delGGT impacting on SARS1 splicing. Disruption of exon 7 donor site leads to the use of an alternative AG donor site located 17–18 bases downstream. This results into the inclusion of 16 intronic bp into the coding sequence, and consequently in the in-frame insertion of 5 amino acids. (C) Primary and secondary structure elements of SARS1. Both sequences are shown starting at F(Phe) 316. WT residues: black; inserted residues: blue. Underlined residues correspond to those being part of the conserved motif 2, present in class II aminoacyl-tRNA synthetases. The two residues in red correspond to F321 and E325 that are involved in ATP and serine recognition, respectively. Below each sequence (letters in grey), secondary structure elements are shown (b=β strand, h=α-helix and -=loop region). (D) Three-dimensional model (based on 4L87, modelled with PyMol) showing the ATP/AMP-Ser binding region of SARS1. Cyan: WT model; dark blue: motif 2; red: mutated region (insertion); pink: AMP-serine analogue. (E) Western blot analysis showing SARS1 and γ-tubulin protein levels in fibroblasts from the patient and age-matched controls (n=3). γ-Tubulin was used as a loading control. (F) SARS1 serylation assays in control and patient-derived fibroblast S100 extracts. tRNA concentration was 22 µM. Data were corrected according to the extract’s concentration. Experiments were performed five times independently.

WES in our patient, followed by variant prioritisation using an in-house bioinformatics pipeline that uses interactome networks based on the clinical presentation (in standard HPO terms),^12^ revealed a candidate heterozygous deletion variant in *SARS1.* The variant (chr1:109778053_109778055delGGT, hg19) causes the ablation of a canonical splice site (c.969_969+2delGGT) in the boundary of exon 7 and intron 7. This variant was prioritised due to: (i) its absence from gnomAD^13^ and other databases of control individuals (ExAC, 1000 Genomes); (ii) being a canonical splice site variant in a predicted loss-of-function intolerant gene and (iii) being located in a gene (*SARS1*) previously associated with similar clinical presentation as our patient (OMIM #617709). After cosegregation analysis in the parents, this variant was found to be *de novo*. Patient fibroblast cDNA sequencing showed that this change causes the inclusion of 16 intronic bp into the cDNA, which results in the in-frame insertion of 5 amino acids (p.Lys323_Ile324insSerArgTrpValArg) (figure 1A, B, online supplemental figure 1).

### Structural and functional characterisation

The deletion is located in evolutionarily conserved motif 2 (residues 291–329) of the *SARS1* aminoacylation domain (online supplemental figure 2). A 3D model analysis showed that the inserted residues perturbed the secondary structure of *SARS1* by disrupting an important β-strand and displacing the side chain of Phe_321_ (figure 1C, D). Residue Phe_321_ is predicted to be important for the recognition of the adenosine ring (A ring) of the AMP molecule through stacking interactions,^14 15^ suggesting that this variant may affect the overall aminoacylation activity of *SARS1*. Furthermore, L-serine binding to residue 325 could also be compromised.^14 15^

In higher eukaryotes, binding of the aminoacylation reaction intermediate analogue (Ser-SA) in the active site induces conformational change and tightening of the tRNA binding domain as well as structural ordering of insertions I and II. These conformational changes are necessary to correctly execute the aminoacylation activity and are likely impaired by this mutation.^14–16^

We next assessed the effects of the variant on protein stability by western blot analysis, using a polyclonal antibody against human SARS1 (Ref: ab183025, Abcam). Results indicate that the insertion did not significantly change SARS1 levels in the patient’s fibroblasts (figure 1E).

To assess SARS1 aminoacylation activity, we performed serylation enzymatic assays using the patient’s fibroblasts compared with sex-matched and age-matched controls (n=3). In brief, we measured radioactivity in fibroblast S100 extracts of the index case and controls at different time points during a 6-hour incubation with ^14^C-radiolabelled L-serine. Five independent assays performed in duplicate were carried out. Mutant fibroblasts showed reduced (~30%) aminoacylation activity (figure 1F). After ACMG/AMP criteria evaluation,^17^ and taking into account both *de novo* segregation (PS2) and enzymatic analysis (PS3), this variant is classified as pathogenic.

### Analysis of hSARS and its mutant in *S. cerevisiae* yeast revealed dominant-negative effects

**Figure 2:**
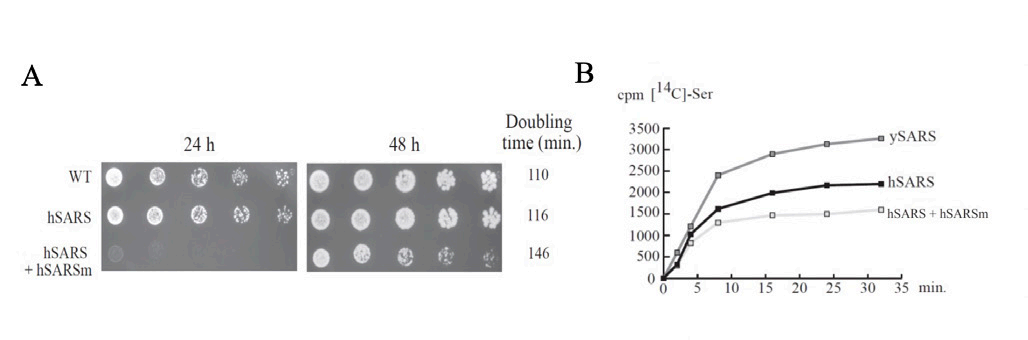
Functional testing using *Saccharomyces cerevisiae* complementation/serylation assays confirm seryl-tRNA synthetase 1 (*SARS1*) mutation’s deleterious effects. (A) Growth analysis of *S. cerevisiae* strains by drop test analysis on SDC-Ura-Leu media. Yeast strain detailed genotypes are detailed in online supplemental table 2. Doubling time of the various strains was determined in liquid SDC-Ura-Leu media in three independent experiments. The results are depicted on the right side. (B) SARS1 serylation assays in *S. cerevisiae* S100 extracts. tRNA concentration was 22 µM. Data were corrected according to the extract’s concentration. Experiments were performed five times independently.

To further study the effect of the mutation, we performed complementation studies in the yeast strain *Schizosaccharomyces pombe*, which harbours a SARS1 homologue (seryl-tRNA synthetase SES1, YDR023w). This is an essential gene, thus a plasmid copy of the SES1 must be provided on deletion to sustain growth of the haploid strain. We thus constructed a centromeric yeast plasmid (pRS316-ySARS) carrying the *URA3* auxotrophic marker and the *SES1* gene, including ORF and promoter regions. Subsequently, the *SES1* ORF was replaced by either human WT SARS1 cDNA (plasmid pRS316-hSARS) or the mutant version of hSARS (plasmid pRS316-hSARSm) (figure 2A).

Spore analysis showed that substituting SES1 for WT human SARS (hSARS) allowed the generation of viable strains, with similar growth capacities figure 2B, indicating that human SARS1 can compensate for SES1 absence. In contrast, the mutant plasmid carrying the insertion identified in our patient (hSARSm), did not allow the generation of viable haploid strains, meaning that mutant hSARS1m is not functional in yeast (online supplemental figure 3). However, when the hSARS strain was cotransformed with a plasmid expressing hSARSm (emulating heterozygosis), an important growth defect was observed, strongly suggesting a dominant negative effect of hSARSm over the WT SARS1 form (figure 2A).

To assess hSARS aminoacylation activity, we performed similar serylation assays as those performed in fibroblasts, using yeast extracts transfected with hSARS, or cotransfected with hSARS+hSARSm. S100 extracts were incubated with ^14^C-radiolabelled L-serine for 30 min. Interestingly, the hSARS+hSARSm strain showed a 30% lower activity than hSARS alone, consistent with previous results in fibroblasts, and again suggesting a dominant negative effect of hSARSm over hSARS (figure 2B).

### Loss of SARS1 activity causes cellular senescence

**Figure 3:**
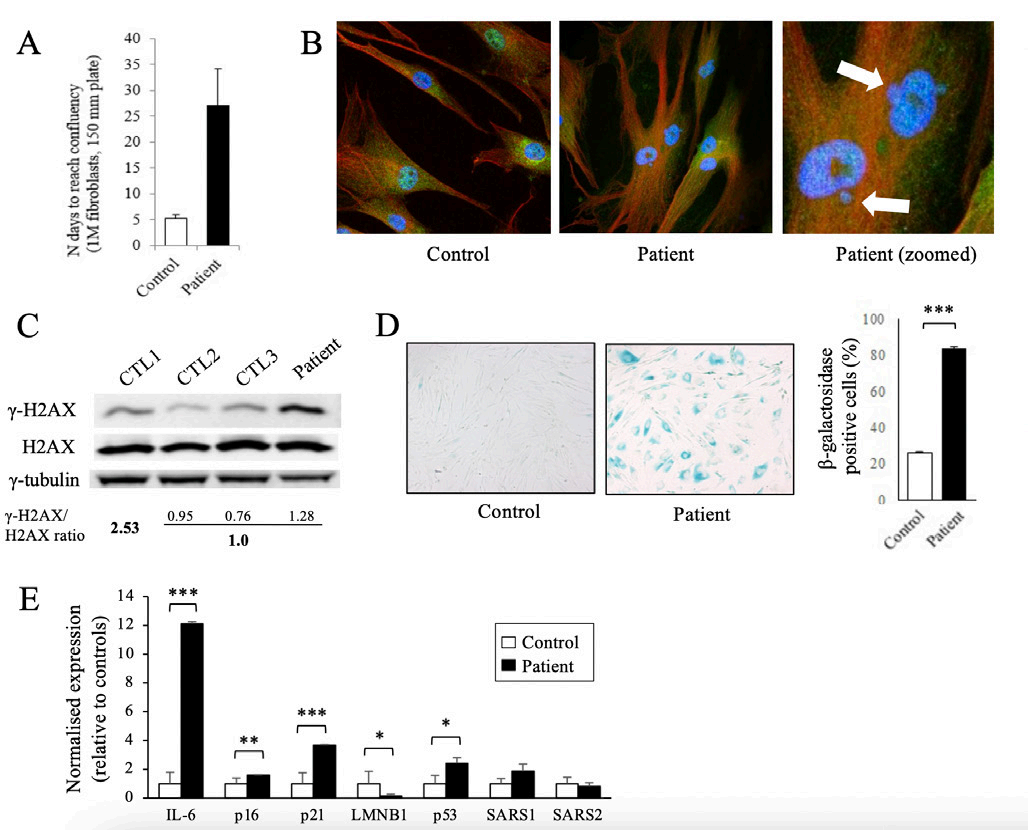
Patient-derived fibroblasts show senescence-related features. (A) Proliferation measurements. Number of culture days needed for 1 million fibroblasts to reach confluency in 150 mm plates. Patient fibroblasts proliferated at a very much slower rate than controls (n=3). Measures were taken thrice. (B) Immunofluorescence images showing seryl-tRNA synthetase 1 (SARS1) localisation (green) in patient (passage 13) and control fibroblasts (n=4, passages 12–15). Blue: DAPI. Red: gamma-tubulin. Patient fibroblasts’ nuclei are structurally affected and micronuclei can be observed (white arrow). Patient fibroblasts also show cell division abnormalities. (C) Western blot analysis showing H2AX and phosphorylated H2AX (γ-H2AX) protein levels in fibroblasts from the patient (passage 13) and age-matched controls. γ-Tubulin was used as a loading control. (D) β-Galactosidase (β-gal) staining in patient (passage 13) and control fibroblasts (n=4, passages 12–15). Positive β-gal cells (in blue) are highly increased in the patient. Left: representative microscopy images. Right: quantification of β-gal-positive cells (%). (E) qRT-PCR results show upregulation of senescence-associated secretory phenotype (SASP) genes in patient’s fibroblasts compared with controls (n=5). SARS1 and SARS2 genes were also quantified. All experiments were performed three times, independently. The values are represented as the means+SD, and Student’s t-test was performed (*p<0.05; **p<0.01; ***p<0.001).

Further functional analysis indicated that the patient’s fibroblasts proliferated very slowly, reaching confluence at least four times slower than controls (figure 3A). Mutant fibroblasts showed abnormal shape, being more enlarged and rounded than controls, with nuclear structural alterations such as micronuclei and abnormal mitosis, suggesting an underlying cell division defect (figure 3B). The distribution of WT and mutant SARS1 was consistent with a previously reported, mainly cytosolic pattern with some nuclear localisation.^8 8^ Moreover, β-gal staining revealed increased senescence in patient’s fibroblasts compared with controls (figure 3D). We thus set out to investigate the main molecular effectors of cellular senescence and DNA damage, such as phosphorylated histone H2AX (γ-H2AX).^18^ Indeed, western blot analysis showed an increase of γ-H2AX levels in patient’s fibroblasts compared with controls (figure 3C). Finally, the mRNA levels of senescence-associated secretory phenotype (SASP) genes such as interleukin-6, p21, p16 and p53 were markedly increased in the patient’s fibroblasts, confirming a senescent phenotype. *SARS1* and *SARS2* mRNA levels did not vary compared with controls (figure 3E).

## Discussion

In this study, WES identified for the first time a *de novo* variant in *SARS1,* challenging the described recessive mode of inheritance, in a patient affected with complex spastic paraparesis with ataxia, seizures and intellectual disability. Two recent articles reported homozygous missense mutations in *SARS1* also in close proximity to the active site (c.514G>A, p.Asp172Asn and c.638G>T, p.Arg213Leu), in two families affected by a neurodevelopmental syndrome including microcephaly, ataxia, seizures, moderate intellectual disability and other anomalies such as cardiomyopathy, deafness and decompensation during fever.^9 10^ Alterations in the central nervous system, such as MRI abnormalities including leucoencephalopathy, ataxia and seizures, have been reported in both cytoplasmic and mitochondrial ARS-related diseases.^3 4 19 20^

In this report, we showed that (i) SARS1 activity was significantly decreased both in patient’s fibroblasts and in the ‘heterozygous’ yeast strain; (ii) mutated *SARS1* protein did not substitute SES1 activity in yeast and (iii) cell growth was impaired both in patient fibroblasts, and in the yeast strain expressing simultaneously WT and mutated SARS1 forms, emulating our heterozygous patient. These results, together with the presence of five healthy *SARS1* loss-of-function variant carriers in the gnomAD database, suggest that this mutation operates through a dominant negative effect, arguing against a haploinsufficiency mechanism. Interestingly, a very recent work described a recurrent *de novo* pathogenic variant in *NARS1*, which produced a protein lacking the ATP-binding domain.^21^ This observation, in addition to our work, strongly suggests that mutations impacting the ATP-binding domain function can yield dominant-negative mutation effects.

In addition, functional characterisation of the patient’s primary fibroblasts indicated severe growth defects and cellular senescence, in combination with a SASP signature. It is intriguing that the opposite mechanism, an overexpression of SARS1 in a transformed BG/HeLa cell line results in increased levels of p21, p16 and β-gal and less telomerase recruitment to telomeres, also leading to cellular senescence,^22^ indicating that a tight regulation of SARS1 levels is pivotal for homeostatic control of cell growth and cell cycle. Thus, SARS1 emerges as the only known tRNA synthetase with such a non-canonical, specific function in cellular senescence. Our results further strengthen the notion that SARS1 dysfunction results into increased senescence levels, and show for the first time how it can be induced by impairing serylation activity, instead of modifying SARS1 expression.

In a more global perspective, tRNA metabolism is closely associated with lifespan and ageing through regulation of processes such as tRNA transcription, modification/transformation of tRNAs, aminoacylation or the effect of the previous on global transcription. As illustrative examples, *C. elegans* models in which two tRNA synthetase genes were downregulated resulted into increased lifespan *(LARS2*,^23^ *LARS1* 24), whereas decreased lifespan was observed in several *Drosophila* models with inactivation or decreased expression of *GARS1*, *MARS2* or *MARS1*.^25^ Along these lines, there is previous evidence connecting precocious senescence and neurodevelopmental phenotypes in the literature, including a developmental delay disorder caused by mutations in *ALKBH8*, a methyltransferase that acts on the wobble uridine base of several tRNA.^26^ Alkbh8-deficient mouse embryonic fibroblasts showed increased β-gal levels, heterochromatin foci and senescence-associated secretory phenotype markers.^21^ Another tRNA methyltransferase gene, *NSUN2*, whose recessive loss-of-function mutations cause intellectual disability (OMIM: 611091) is also associated with senescence: *NSUN2*-knockdown human fibroblasts showed repressed cell proliferation and accelerated replicative senescence.^27 28^

In summary, we report a novel dominant-negative mutation in *SARS1* and a novel mode of inheritance, underscoring the need for caution in interpreting variants in ARS enzyme genes according to inheritance modes, with important implications for genetic diagnosis and counselling. Furthermore, we unveil pivotal roles of this enzyme in the control of cell growth and senescence, underlying the neurodevelopmental phenotypes of affected patients.

## Data Availability

All data relevant to the study are included in the article or uploaded as supplementary information.
