# Supplementary material for: Loss of seryl-tRNA synthetase (SARS1) causes complex spastic paraplegia and cellular senescence
Source: J Med Genet. 2022 Aug 30;59(12):1227–33. doi: 10.1136/jmg-2022-108529 (PMC9691831; doi:10.1136/jmg-2022-108529)

SUPPLEMENTAL MATERIAL

Supplemental Table 1: RT-qPCR and RT-PCR primers

IL-6<br>(RT-qPCR)	Forward: AGTCCTGATCCAGTTCCTGC<br>Reverse: CTACATTTGCCGAAGAGCCC
p16<br>(RT-qPCR)	Forward: CTGAGGAGCTGGGCCATC<br>Reverse: GCAGTTGTGGCCCTGTAG
p21<br>(RT-qPCR)	Forward: CTGGAGACTCTCAGGGTCGAA<br>Reverse: CCAGGACTGCAGGCTTCCT
p53<br>(RT-qPCR)	Forward: AAGAAACCACTGGATGGAGAA<br>Reverse: CAGCTCTCGGAACATCTCGSS
LMNB1<br>(RT-qPCR)	Forward: AAGCAGCTGGAGTGGTTGTT<br>Reverse:TTGGATGCTCTTGGGGTTC
SARS1<br>(RT-qPCR)	Forward: CCACTACCCGTACCATCTGC<br>Reverse: CTGCCCTCATGTTGCTTCTT
SARS2<br>(RT-qPCR)	Forward: CCTTCTCATCGCGCTCCT<br>Reverse: TGTGGGTGGGTTCTTAGCTT
H36B4<br>(RT-qPCR)	Forward: ACGGGTACAAACGAGTCCTG<br>Reverse: GCCTTGACCTTTTCAGCAAG

SARS1<br>(RT-PCR)	Forward: GGAACAGGCTCTCATCCAGTA<br>Reverse: AGGAGACCAACTCACGGAAG

Supplemental Table 2: Yeast strains used in this study.

Name	Genotype	Source
Y23962	MATa/MATα; ura3Δ0/ura3Δ0; leu2Δ0/leu2Δ0; his3Δ1/his3Δ1; met15Δ0/MET15; LYS2/lys2Δ0; YDR023w/YDR023w::kanMX4	EUROSARF
hSARS shuffle	MATa; ura3Δ0; leu2Δ0; his3Δ1;YDR023w::kanMX4 + pRS316-hSARS	This study
WT	MATa; ura3Δ0; leu2Δ0; his3Δ1;YDR023w::kanMX4 + pRS316-ySARS + pRS315	This study
hSARS	MATa; ura3Δ0; leu2Δ0; his3Δ1;YDR023w::kanMX4 + pRS316-hSARS + pRS315	This study
hSARS + hSARSm	MATa; ura3Δ0; leu2Δ0; his3Δ1;YDR023w::kanMX4 + pRS316-hSARS + pRS315-hSARSm	This study

Supplemental Figure 1: RT-PCR and cDNA sequencing results from fibroblasts for SARS1-mutated patient and a control. A: Agarose gel electrophoresis image showing RT-CR products. B: Sanger sequencing results of patient and control RT-PCR products. C: Sanger sequencing results of WT/mutated sequences after cloning patient's cDNA PCR products into a pCMV-Tag2 plasmid.

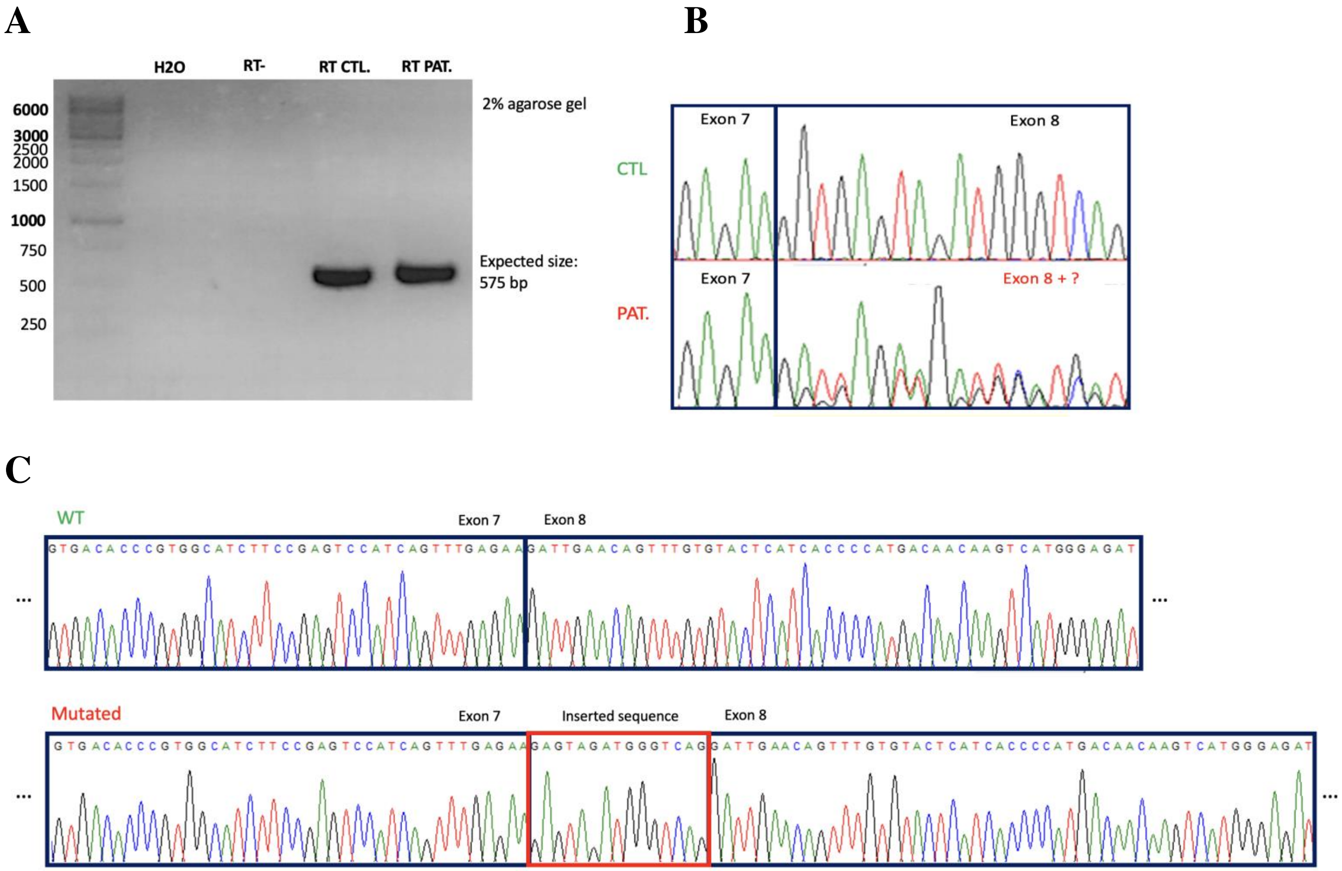

Supplemental Figure 2: Conservation of mutation site in SARS1. p.Lys323_Ile324 insSerArgTrpValArg insertion is located in an evolutionary conserved region of SARS1 protein. Motif 2 (291-329 aa) is colored in yellow, and contains active site residues important for SARS1 aminoacylation (in boxes).

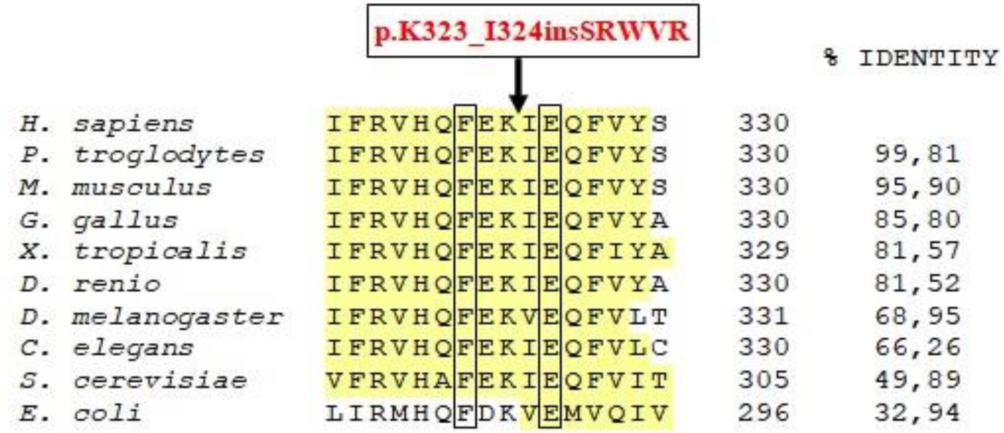

Supplemental Figure 3: Diploid *S cerevisiae* yeast strains used for complementation assays. Tetrad analysis of haploid spores showed that haploid strains containing pRS316-hSARSm (carrying the SARS1 mutation) did not grow.

.

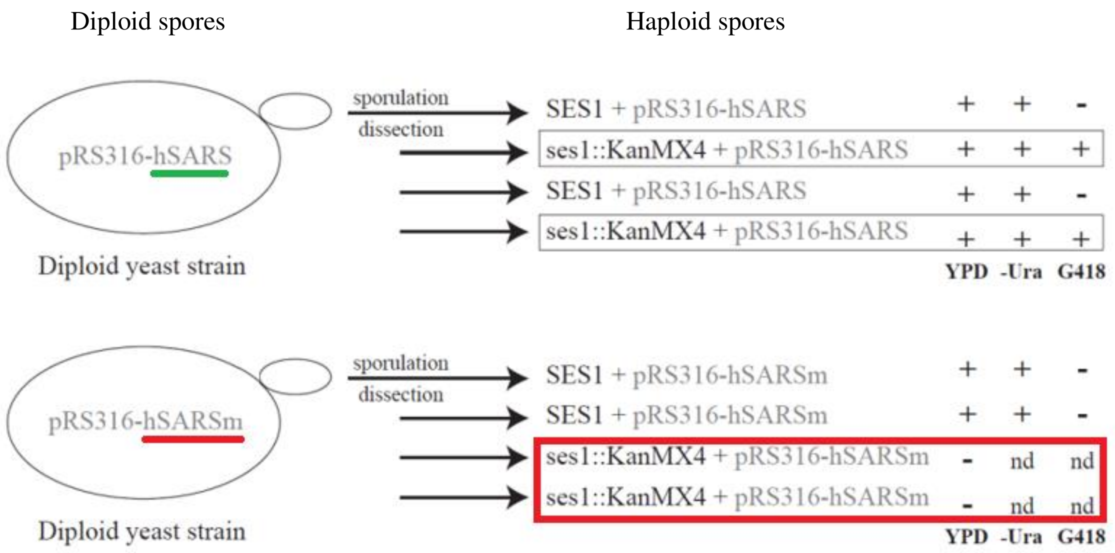

Supplement: Supplementary data [file jmg-2022-108529supp001.pdf]
